# Goal-directed unequal attention allocation during multiple object tracking

**DOI:** 10.3758/s13414-019-01674-y

**Published:** 2019-02-13

**Authors:** Emily M. Crowe, Christina J. Howard, Angela S. Attwood, Christopher Kent

**Affiliations:** 1 School of Psychological Science, University of Bristol, 12a Priory Road, Bristol, BS8 1TU UK; 2 Department of Psychology, Nottingham Trent University, Nottingham, UK

**Keywords:** Attention, Multiple object tracking, Unequal attention splitting, Target priority, Goal-directed

## Abstract

In standard multiple object tracking (MOT) tasks the relative importance of the targets being tracked is equal. This is atypical of everyday situations in which an individual may need to prioritize one target relative to another and so allocate attention unequally. We report three experiments that examined whether participants could unequally split attention using a modified MOT task in which target priority was manipulated. Specifically, we examined the effect of priority on participants’ magnitude of error and used a distribution mixture analysis to investigate how priority affected both participants’ probability of losing an item and tracking precision. Experiment 1 (trajectory tracking) revealed a higher magnitude of error and higher proportion of guessing for low- compared with high-priority targets. Experiments 2 (trajectory tracking) and 3 (position tracking) examined how fine-grained this ability is by manipulating target priority at finer increments. In line with Experiment 1, results from both these experiments indicated that participants could split attention unequally. There was some evidence that participants could allocate attention unequally at fine increments, but this was less conclusive. Taken together, these experiments demonstrate participants’ ability to distribute attention unequally across multiple moving objects but suggest some limitation with the flexibility of attention allocation.

## Introduction

Allocating attention to multiple objects as they move around the environment is required for both everyday activities (e.g., driving a car and playing team sports) and real-world occupations (e.g., air traffic control and CCTV monitoring). This ability has been extensively studied using the multiple object tracking (MOT) task (Pylyshyn & Storm, 1988). In this task, several objects are presented on screen, a subset of which are temporarily identified as targets. Participants then track the targets amongst visually similar distractors as they move randomly around the screen. At the end of a trial, all objects stop moving and participants are queried about the status (i.e., target or distractor) of an object, the trajectory of a target, or the position of a target. Typically, participants can simultaneously track approximately four objects (e.g., Intriligator & Cavanagh, 2001; Pylyshyn & Storm, 1988; Scholl, Pylyshyn, & Feldman, 2001). Tracking performance is limited by factors such as the number (Yantis, 1992), speed (Alvarez & Franconeri, 2007), and spacing (Franconeri, Lin, Pylyshyn, Fisher & Enns, 2008; Tombu & Seiffert, 2008) of objects. Such limits on tracking indicate that there is a finite attentional resource because tracking performance deteriorates as the number of targets increases (e.g., Alvarez & Franconeri, 2007; Franconeri et al., 2008; Yantis, 1992). The structure of this resource is debated, with some authors proposing a fixed architectural system consisting of a limited number of discrete pointers or slots (e.g., Pylyshyn, 1989) and others postulating a more flexible, continuous pool of resources (e.g., Alvarez & Franconeri, 2007).

Fixed theories emerged following the consistent finding that approximately four targets could be accurately tracked in MOT tasks. Pylyshyn’s (1989) Fingers of Instantiation (FINST) model consists of a fixed set (i.e., three, four, or five) of indexes or slots that can be assigned to objects to provide a connection between the outside world and visual representations in cognition. Cavanagh and Alvarez’s (2005) multifocal theory posits that multiple foci of attention, rather than visual indexes in the FINST model, track each object. These two models suggest that tracking limitations are due to fixed architectural constraints, namely the number of visual indices or attentional foci.

Flexible resource theories suggest that there is a continuous pool of the attentional resource that can be drawn upon for tracking multiple objects. Alvarez and Franconeri (2007) proposed the FLEX model (FLEXibly allocated indexes), which suggests that objects are tracked by flexible indexes (FLEXes), the total number of indexes is limited by the finite resource. The limit on tracking is set by this shared resource that determines the resolution of each FLEX such that when fewer items are tracked, the tracking resolution is higher, consistent with findings relating to spatial precision of target representations (Howard & Holcombe, 2008; Howard, Masom, & Holcombe, 2011). Kazanovich and Borisyuk (2006) proposed that tracking is accomplished by a set of central oscillators that synchronize with each other to label objects in the focus of attention. Tracking is limited by the phase space of such oscillators and so tracking is better with fewer independent oscillators that are more sparsely distributed in phase space. Franconeri et al. (2010) proposed the spatial interference theory of MOT that suggests that the constraints on tracking are determined by the spatial relationship between targets and distractors (i.e., objects that participants do not have to keep track of). This alternative to the FLEX model suggests tracking errors are the result of distractors or other targets entering the inhibitory surround (i.e., a spatial region) of targets (Meyerhoff, Papenmeier & Huff, 2017).

A parallel debate persists in the visual short term-memory (VSTM) literature in which a capacity limit of 3–5 items has often been reported (Cowan, 2001). Such findings have led to the proposal of fixed, slot-based theories of VSTM that suggest that, irrespective of the complexity of objects, only a limited, fixed number of items can be stored (e.g., Awh, Barton & Vogel, 2007; Luck & Vogel, 1997). Other authors (e.g., Alvarez & Cavanagh, 2004; Eng, Chen, & Jiang, 2005) propose that the number of objects that can be stored is more flexible and determined by the complexity of objects. Distinguishing between fixed and flexible mechanisms underlies a variety of questions within cognitive psychology that are inherently related. One closely related task is multiple identity tracking (MIT) in which participants must maintain information about the identity of multiple objects as they move (critically in MOT tasks the features of objects are identical, whereas in MIT each tracked object has a unique feature to identify it; Oksama & Hyönä, 2004). Oksama and Hyöna (2008) suggest that, in some MIT tasks, target-relevant location information is stored in VSTM and therefore tracking limits are derived from the structure of the resource underlying VSTM. Characterizing the mechanisms that underlie tracking is therefore important to provide a greater understanding of other, related cognitive processes as well.

Both fixed and flexible theories are based on results from experiments using assumed equal attention splitting. Under either the fixed or flexible theories, unequal attention splitting, in which objects for tracking are allocated different amounts of the attentional resource, is theoretically possible. As an analogy to help distinguish the two accounts, water can be used to represent the attentional resource underlying tracking. Under a fixed account, water takes the solid form of ice cubes and so the fixed number of ice cubes or slots can be unequally distributed across objects in only a limited number of ways (i.e., attention slots could be split between two targets according to a limited number of ratios: 4:0; 3:1; 2:2 or 5:0; 4:1; 3:2). In contrast, under a flexible account water takes the liquid form and so can be flexibly allocated unequally in any way (e.g., 37% : 63%). It is important to recognize that the structure of the attentional resource could fall anywhere between these two points and so a key question, addressed here, is *how* flexible the resource is.

Previous studies have demonstrated stimulus-driven unequal allocation. Liu et al. (2005) modified the typical MOT task so that half the objects moved at 1 °/s and the other half at 6 °/s. There was no difference in tracking performance between fast- and slow-moving targets indicative of unequal attention allocation. More specifically, more of the resource could have been allocated to the faster (more demanding) target, which resulted in similar tracking accuracy across both speed conditions. Chen, Howe, and Holcombe (2013) compared the speed limits at which participants could track a critical target when the second target was moving at either the same or a slower speed. The speed limit for the critical target was higher if the second target was moving slow rather than fast. This suggests that participants allocated attention unequally with more attention available to allocate to the fast-moving target when the secondary target was moving slower. Together, these results provide evidence consistent with participants’ ability to unequally allocate attention in a stimulus-driven manner.

Some authors have also examined participants’ ability to shift attention on-line (i.e., during a trial). Iordanescu, Grabowecky, and Suzuki (2009) argued that targets in crowded situations (i.e., those in danger of being mistaken for distractors) were localized more precisely than uncrowded targets, suggesting that more attention was allocated to these “high-risk” targets. This supports the notion of unequal attention allocation and, additionally, suggests that the attention allocated to a given target can be changed during tracking. Nevertheless, this result should be interpreted with some caution because proximity (to the nearest distracter) was not manipulated directly (i.e., object trajectories were randomly determined) and, therefore, other display characteristics could have been affected as well as proximity (Chen, Howe, & Holcombe, 2013; see also contradictory findings by Howard, Masom, & Holcombe, 2011). Howe et al. (2010) adapted the “simultaneous-sequential paradigm” (Eriksen & Spencher, 1969) to examine whether attention could be reallocated between targets during tracking. In the simultaneous condition, all objects moved and paused simultaneously, whereas in the sequential condition objects were divided into two groups and moved alternatively. There was no difference in tracking performance between objects in the simultaneous and sequential conditions, which suggests that participants could not reallocate attention unequally between targets during tracking. Meyerhoff, Schwan, and Huff (2018) conducted a series of experiments to explore whether inter-object spacing guides visual attention. A bias towards temporarily close objects (both in term of spatial attention allocation and eye movements), which persisted even when the bias was harmful for the task, was observed indicating both unequal attention allocation and updating of attention allocation during a trial (see also Zelinsky, & Todor, 2010). In other work Meyerhoff, Papenmeir, Jahn, and Huff (2016) revealed that such unequal allocation of the attentional resource in a stimulus-driven manner is advantageous to avoid confusion between targets and close distractors indicating that attention can be flexibly allocated during tracking.

Goal-directed unequal attention allocation in MOT has also been documented. Cohen, Pinto, Howe, and Horowitz (2011) modified the instructions given to participants in an MIT task. In one condition, participants were instructed to prioritize the locations over the identities of target and, in another, were instructed to place equal emphasis on both location and identity information. Position-tracking performance was higher when prioritization instructions were given demonstrating unequal attention allocation between the location and identity information associated with the same target. However, to our knowledge, no research has addressed whether participants can split attention unequally between distinct targets in a goal-directed manner (i.e., not to different features of the same object). Examining the way in which participants can split attention unequally in a strategic manner has the potential to inform the debate regarding the structure of the attentional resource underlying tracking because the amount of attention allocated to a given object can be directly manipulated. This allows examination of the resource-versus-performance function, the shape of which would be different for fixed and flexible theories. As well as being theoretically important, unequal allocation of attention is highly relevant to the real-world in situations where one wishes to prioritize, and so allocate more attention to one target over another target, which nonetheless needs tracking.

Yantis (1992) showed goal-directed attention allocation within an MOT framework. Participants who were instructed to group all targets together displayed higher tracking accuracy than those who were given neutral tracking instructions. This shows that participants modified their tracking strategy in a goal-directed manner. Brockhoff and Huff (2016) combined a typical MOT task with a non-interfering top-down identification task. Participants were instructed to identify the behavior of dynamic cartoon eyes. The cartoon eyes were the objects in the MOT task and the moving pupils cued either a single target or single distractor by all rotating to look towards that specific object. Participants could ignore or prioritize objects based on cueing, thus indicating goal-driven attention allocation during the MOT task. Taken together, these results demonstrate top-down mechanisms driving attentional allocation but do not provide any insight into the potential for top-down *unequal* attentional allocation between two simultaneously tracked objects within a trial.

Goal-directed unequal attention allocation has been demonstrated in other attention-based tasks in which participants are instructed to allocate different proportions of their attention accordingly. Miller and Bonnell (1994) instructed participants to pay a certain amount of attention to a line-length discrimination task on the left side of the screen and the remaining attention to the right side and revealed that sensitivity increased with the proportion of attention devoted to that side. Fitousi (2016) instructed participants to allocate differential amounts of their attention to the top and bottom halves of a face. Such instructions were effective in modifying the amount of attention allocated to either half of the face, with participants’ performance improving as a function of attention allocation (Fitousi, 2016). Atkinson, Berry, Waterman, Baddeley, Hitch, and Allen (2018) used probe frequencies (i.e., how frequently a more valuable item was tested) to examine whether memory for an item was enhanced if participants were told it would be tested more frequently. Memory was enhanced for the relatively more valuable item, indicating that attention can be directed according to probe frequencies. However, on the contrary, Chen, Howe, and Holcombe (2013) claim that it would be difficult to induce participants to allocate a specific proportion of attention to two targets during an MOT task due to the extended duration of tracking across an MOT trial. We empirically test this claim here.

The present series of experiments examined whether participants could split attention unequally to multiple moving objects in a goal-directed manner. We used modified MOT tasks in which the priority of targets was manipulated to examine the effect of target priority on tracking performance. Such modification resulted in the task encompassing components of both MOT and MIT. MIT requires participants to maintain location-identity bindings during tracking (Mayerhoff, Papenmeier, & Huff, 2017). This modified MOT task requires participants to assign a priority (i.e., an identity) to each target during a trial and therefore fits with an MIT task. However, the index of tracking performance fits more closely with the MOT literature because the targets’ position or trajectory is queried rather than an identity-related response.

Experiment 1 examined whether participants could split attention unequally between high- and low-priority targets. Experiments 2 and 3 explored how fine-grained participants’ ability to allocate attention unequally was by manipulating the target priorities at finer increments. Tracking performance was measured as the absolute error between the actual and estimated trajectory (Experiments 1 and 2) or location (Experiment 3). In addition, we used a mixture distribution analysis (based on Zhang & Luck, 2008) to estimate the precision of tracking and the guessing rate. We hypothesized that the magnitude of tracking error, proportion of guessing, and the precision of tracking would be lower for the higher priority targets in all three experiments indicative of strategic unequal attention allocation.

## Experiment 1

### Method

#### Participants

Twenty-seven undergraduate students from the University of Bristol participated in return for course credit. G*Power version 3.1 (Faul, Erdfelder, Lang, & Buchner, 2007) was used to calculate sample size for all experiments. Due to institutional constraints, we over-recruited for all experiments. Based on existing data from our lab suggesting an effect size of *dz* = 0.73, this sample size gave us at least a 95% chance of observing a similar effect size, with alpha set at .05 for two-tailed tests.

#### Design

Target priority was manipulated in a within-subject design with three levels: low (25%), equal (50%), and high (75%), which reflected the veridical probability of a target being queried over the course of the whole experiment. The primary dependent variable was magnitude of angular error, indexed by the degree of error from the queried target’s actual trajectory (i.e., the direction it was heading in) to the participant’s reported trajectory at the end of the trial. For example, if at the final moment of the moving tracking display, the queried target was last moving upwards and rightwards at an angle of 10° clockwise from vertical, and the participants reported that it was moving directly upwards, then this would constitute a magnitude of angular error of 10°. The proportion of guess trials and precision of representations, calculated from the mixture modelling analysis were also dependent variables.

#### Procedure

Stimulus displays were presented on a 17-in. CRT monitor with a resolution of 1,024 x 768 pixels and a refresh rate of 85 Hz. Viewing distance was approximately 40 cm. Participants completed the task in a dimly lit room. A custom made program was written using MATLAB version 2014b (The MathWorks, Inc, 2014) and the Psychtoolbox extensions (Brainard, 1997; Kleiner et al., 2007; Pelli, 1997).

**Fig. 1:**
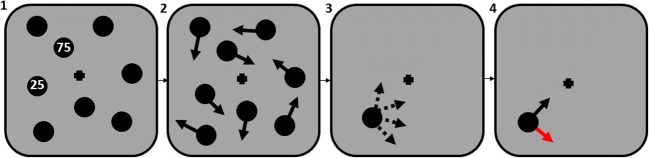
Trial timeline. (**1**) Eight discs were presented on screen. Discs containing a value inside denote the likelihood of that target being queried at the end of a trial. (**2**) All discs moved around the screen. (**3**) All discs except one disappeared. Participants estimated what direction the disc was heading it at the end of the trial using a rotatable pointer. (**4**) Participants were given feedback. A second arrow was presented that indicates the correct target trajectory. If a participant’s trajectory estimate was within 12.5° of the correct trajectory, the arrow turned green; otherwise, it turned red

Figure 1 shows a timeline of one MOT trial. On each trial, participants fixated a central black fixation cross and eight black discs with a radius of 1.14° of visual angle, two targets and six distractors, were presented on a mid-gray screen at the start of each trial for 2,000 ms. Each target had one of three numbers (25, 50, 75) presented on them denoting the likelihood of this target would be queried at the end of a trial and so indicating the relative importance of each target (i.e., the 75 and 25 targets were of high and low priority, respectively). On any given trial, the combined values totalled 100. Participants were given clear instructions and the opportunity to ask questions regarding how to allocate their attention before starting the practice trials. The discs then moved randomly around the screen at an average speed of 15.8° per second for between 5,000 and 8,000 ms (randomized for each trial) and underwent perfectly elastic collisions whenever they collided with the edge of the display or another disc. At the end of the trial, all discs disappeared except one of the targets, which remained on the screen. Participants clicked inside the target to activate it which caused a Iine, 1.14° long, to extend from the target’s center. The direction of the line was determined by the position of the participant’s mouse click. Participants then moved the line (using the mouse) to report the target’s trajectory and clicked to confirm their answer. Feedback, consisting of an arrow indicating the correct direction of heading, was given on each trial for 2,000 ms, after which the next trial was presented. Participants completed ten practice trials followed by 250 experimental trials, the order of which was randomized, in ten blocks. The experiment lasted approximately 1 h.

### Results and discussion

One participant was excluded due to their very high magnitude of error (and the model-based analysis suggested they had a very high rate of guessing). Linear mixed effects models (LMEs) were used to analyze the data using the lme4 package (Bates, Mächler, Bolker, & Walker 2014) for the R computing environment (R Development Core Team, 2014). Target priority was entered into the model as a fixed effect. As random effects, there was a random intercept for subjects and a by-subject random slope for the effect of target priority. P-values were obtained by likelihood ratio tests of the full model including terms related to priority against the model without priority included. *Post hoc* comparisons were conducted by comparing the slopes between two adjacent target priorities.

**Fig. 2:**
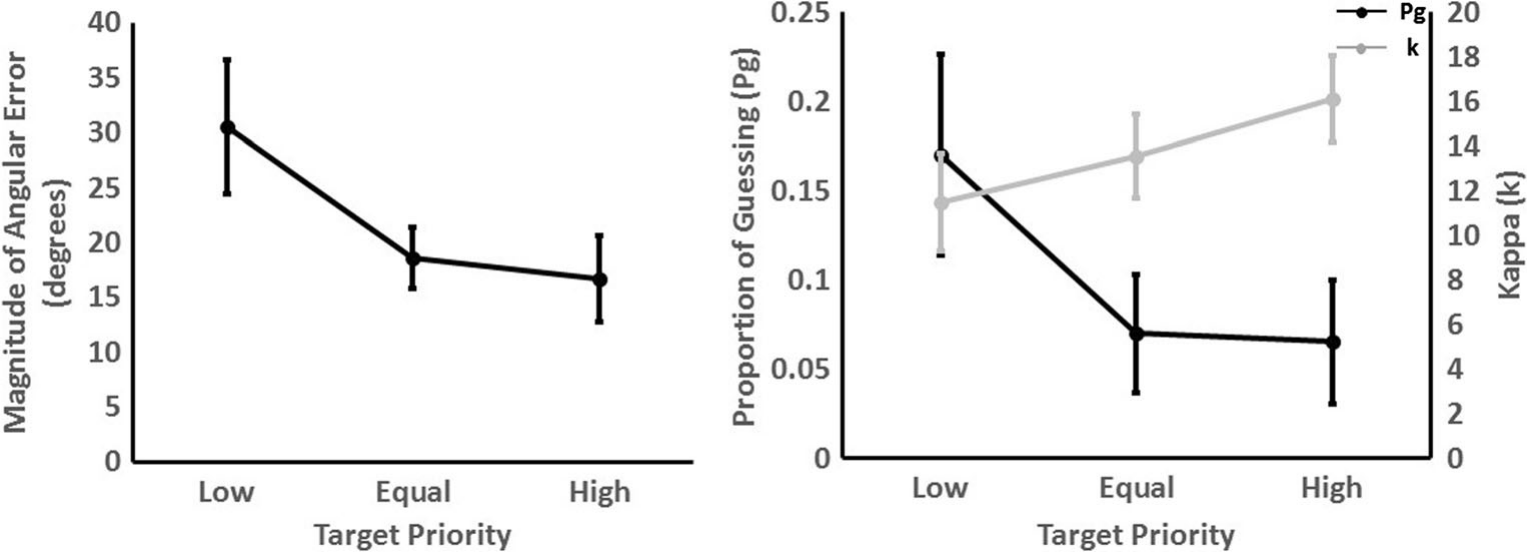
Mean magnitude of error, proportion of guessing and precision of tracking for each target priority in Experiment 1. Error bars represent 95% within-subject confidence intervals using Morey (2008)

There was a main effect of priority, χ2 (2) = 16.60, *p* < .001, whereby the magnitude of angular error decreased as target priority increased, *b* = -0.277, SE = 0.06, *t* = 4.42. *Post hoc* tests showed that there was a higher magnitude angular error in the low-priority than in the equal-priority condition (*b* = -0.48, *t* = 3.53, *p* = .006), but no difference between the equal- and high-priority conditions (*b* = -0.08, *t* = 1.65, *p* = .236) (see Fig. 2, left panel).

It is possible to interpret the distribution of error magnitudes in order to examine the data further. This analysis distinguishes contributions from two sources to differences in overall accuracy. The source is the guessing rate, where guesses may be due to participants’ losing track or otherwise completely withdrawing attention from a target. The second is the precision of representations (due to the amount of allocated attention) of targets^1^. Analyzing data from a series of MOT experiments in which participants judged the heading of a target object, Horowitz and Cohen (2010, following Zhang & Luck, 2008) used a mixture of a uniform distribution (representing the situation where a target is lost and participants must guess) and von Mises (the circular equivalent of the normal distribution, representing the situation where participants have successfully tracked a target, but with varying precision, as reflected in the spread of the distribution). Under a pure slot-based model the precision should not change as set size increases to any level (since a fixed number of slots are allocated, and targets that are not tracked are guessed, which is captured under the uniform guessing distribution). Flexible accounts predict that precision should decrease as the number of items increases for any set size increase. Horowitz and Cohen also tested two hybrid models (again following Zhang & Luck, 2008): the slots + resources model (a fixed number of slots, but a resource that can be unequally allocated among those slots) and the slots + averaging model (a fixed number of slots, but slots can be applied to more than one target if below capacity). Both hybrid models make the same prediction, however: If the number of targets to track is below capacity the precision will decrease as the number of targets increase (either because resources are spread more thinly, or because slots cannot be shared) and asymptote if capacity is reached (as additional targets are not tracked and are guessed, which is captured under the uniform guessing distribution).

**Fig. 3:**
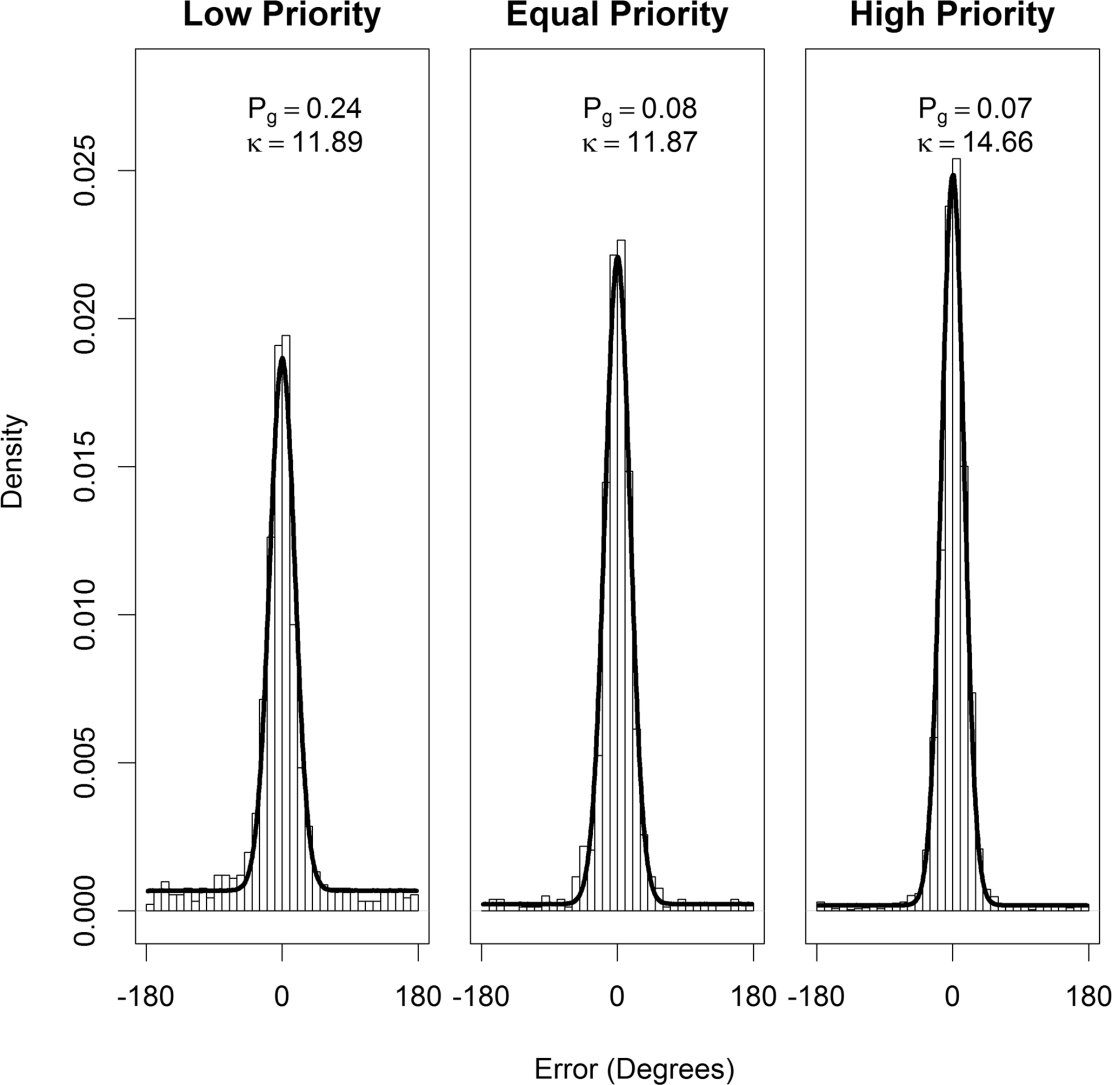
Mixture model fits for the combined data across participants for Experiment 1 for each level of target priority. The density plot displays the actual data and the black line shows the model fit. The proportion of guessing (*P*_*G*_) and precision of tracking (*κ*_*VM*_) parameters are also detailed

In line with the method used by Horowitz and Cohen (2010), we fit a mixture of a uniform circular distribution and von Mises distribution to each participants’ data for each level of priority. We used the fitdistr function from the MASS package (Venables & Ripley, 2002) with von Mises and uniform distributions functions from the “circular” package (Agostinelli & Lund, 2017). The uniform circular distribution, representing the situation where a participant makes a guess response, generates a random value between -180 to 180. The von Mises distribution, representing the situation where a participant has tracked a target, but to a varying degree of precision, is controlled by two parameters: *μ* (the mean) and *κ* (the concentration parameter, which determines the spread of the distribution). The mixture of guessing and tracked errors was controlled by *P*_*G*_, the proportion of guessing. The error distribution, *ε*, is therefore:

1 $$ \varepsilon ={P}_G{f}_{uc}\left(-\mathrm{180,180}\right)+\left(1-{P}_G\right){f}_{VM}\left(\mu, \kappa \right) $$

in which *f*_*uc*_ is the uniform circular distribution function and *f*_*vm*_ is the von Mises distribution function. In our analysis (following Horowtiz & Cohen, 2010) we fixed *μ* = 0 (i.e., average error was zero). We used R (R Core Team, 2015) to estimate *κ* and *P*_*G*_ values via maximum likelihood estimation function fitdistr from the MASS package (Venables & Ripley, 2002) with von Mises and uniform distributions functions from the “circular” package (Agostinelli & Lund, 2017). The mixture model fits (for data combined across participants), for each level of target priority, are shown in Fig. 3. A higher precision value, *κ,* indicates a more leptokurtic distribution which demonstrates higher precision. Therefore, a higher precision value indicates higher precision.

The *κ* and *P*_*G*_ values, estimated for each participant and each level of priority, were then entered into an LME analysis, in an identical manner to the treatment of the magnitude of angular error scores. There was an effect of target priority on the proportion of guessing, χ2 (2) = 11.10, *p* = .004. Participants demonstrated less guessing for high-priority targets, *b* = -0.002, SE = 0.001, *t* = 3.52. There was evidence for a higher proportion of guessing in the low-priority compared to the equal-priority condition, (*b* = -0.004, *t* = 0.32, *p* = .021). However, there was no difference in the proportion of guessing in the high compared with the equal condition, (*b* = -0.004, *t* = 1.65, *p* = .950) (see Fig. 2, right panel).

Finally, there was an effect of target priority on the precision of representations (*κ*), χ2 (2) = 10.52, *p* = .005, with the precision increasing as target priority increased, *b* = -0.09, SE = 0.03, *t* = 3.42 (see Fig. 2, right panel). *Post hoc* tests showed that there was no difference in precision between any of the adjacent levels of target priority (both *t* < 2.12 , *p* > .127).

Experiment 1 showed that participants guessed the trajectory of the low-priority target more frequently than both the equal- and high-priority target. Howard, Rollings, and Hardie (2017) showed that participants’ attention to a target’s position and attention to its motion characteristics are distinct. Therefore, it cannot be assumed that all guess trials were associated with participants having no attention on that target. However, it could be argued that the higher proportion of guessing for low-priority targets indicates participants could not split attention unequally and, therefore, sometimes either lost the target completely (i.e., dropped the target) or confused it with a distractor (i.e., swapped the target with a distractor). However, this is likely an infrequent occurrence given the relatively low proportion of guessing (the majority of trials not modelled as involving a guess response) and a relatively good level of tracking accuracy (indexed by the magnitude of angular error) for the low-priority target. This indicates that some attention was allocated to the low-priority target but, in some cases, this was not sufficient to support updating of a target’s trajectory, which resulted in an increase in guessing.

The effect of target priority on magnitude of error and precision shows that differential amounts of attention were allocated to the high- and low-priority targets, respectively, indicative of unequal attention allocation. This suggests some flexibility with regard to the attentional resource underlying tracking. Specifically, more attention is allocated to the high-priority target, which leads to lower magnitude or error and higher precision. This finding does not fit with slot-based accounts of attention allocation, which would predict that the magnitude of angular error and precision of representations would remain constant because each target is allocated one slot. Flexible and hybrid models can, however, account for these findings because under their assumptions attention is unequally distributed between the two targets resulting in differences in the three indexes of tracking accuracy.

This experiment does not provide insight into how fine-grained this ability is. The extent to which attention splitting is fine-grained refers to the precision with which a division of attention is possible, in an analogous fashion to the way that liquid water makes splitting infinitely more fine-grained than crushed ice or ice cubes. Experiment 2 therefore examined whether participants can split their attention unequally across two targets with smaller disparities in their priority (e.g., 40% vs. 60%) than used in Experiment 1 (i.e., 25% vs. 75%). Exploring the extent to which attention is fine-grained has the potential to distinguish between different models of MOT. For example, under slots + averaging models, each target could be assigned more than one slot and, therefore, unequal attention splitting is theoretically possible. However, in slot + averaging models, attention can only be split unequally in a finite number of ways (i.e., 4-0; 3-1; 3-2). In contrast, under flexible accounts, there is an unlimited number of ways that attention can be split.

## Experiment 2

Experiment 2 further investigated to what extent participants can finely split their attention unequally across multiple moving objects. We manipulated the target priorities at finer increments (70, 60, 50, 40, and 30) than Experiment 1 to enable investigation of the precision with which participants could allocate a pre-specified amount of the attentional resource to a given target. We conducted two identical studies, but one was completed in a single-participant testing environment (i.e., each participant completed the study alone) and another was completed in a group testing environment (i.e., participants completed the study in a group of approximately 20 participants). The study aims and hypothesis were preregistered on the Open Science Framework and can be accessed at: https://osf.io/s5c6h/?view_only=ed239e4a584744249dd1bb53b4742e53 and https://osf.io/ety5r/?view_only=75f6816e4ade4956a3cdfff270190ca5. For brevity and power, we present the combined data from these studies.^2^

### Method

#### Participants

Seventy-nine undergraduate students from the University of Bristol participated in return for course credit (single testing = 36 participants; group testing = 43 participants). Based on existing data from our lab suggesting an effect size of *dz* = 0.54, we powered for a similar effect size of *d* = 0.5, which gave us at least an 80% chance of observing a similar effect size, with alpha set at .05, based on two-tailed tests, for each independent method of testing (i.e., single and group testing power calculations were calculated separately)

#### Design

Target priority was manipulated in a within-subject design with five levels: very low (30), low (40), equal (50), high (60), and very high (70), and reflected the true likelihood of a target being queried over the course of the whole experiment. The dependent variables were the same as in Experiment 1.

#### Procedure

The procedure was identical to that used in Experiment 1 apart from, when providing their response, participants had to indicate whether they thought they were tracking the queried target at the end of the trial or not by clicking the left mouse button for “tracked” and the right mouse button for “not tracked” (labels were put on the mouse buttons) .^3^ This click also activated the response indicator line. Participants then used the same mouse button to finalize their response, as detailed in Experiment 1. In the group testing experiment stimuli were presented in a 1,024 x 768 pixels window of a 21-in. LCD monitor (1,920 x 1,080 resolution) with a refresh rate of 60 Hz.

### Results and discussion

Two participants were removed from the analysis because their overall magnitude of error was very high (and the model-based analysis suggested they had very high levels of guessing). The LME analysis and *post hoc* comparisons used were identical to Experiment 1.

**Fig. 4:**
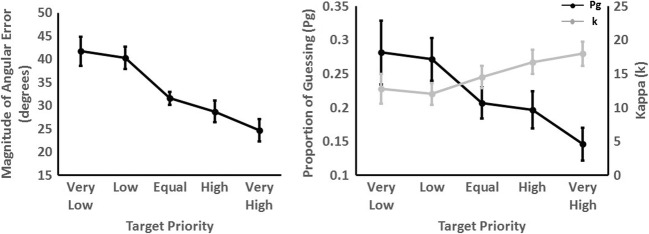
Mean error in magnitude of error, proportion of guessing, and precision of tracking for each target priority in Experiment 2. Error bars represent 95% within-subject confidence intervals using Morey (2008)

There was an effect of target priority on the magnitude of angular error, χ2 (2) = 121.49, p < .001, which decreased as target priority increased, *b* = -0.467, SE = 0.04, *t* = 12.20 (see Fig. 4, left panel). *Post hoc* tests showed no difference in the magnitude of angular error between the very low- and low-priority conditions (*b* = -0.13, *t* = 0.14, *p* = .639). Magnitude of angular error was higher in low compared with equal (*b* = -0.90, *t* = 6.49, *p* < .001), equal compared with high (*b* = -0.29, *t* = 2.50, *p* = .049), and high compared with very high priority conditions (*b* = -0.42, *t* = 5.40, *p* < .001), respectively.

**Fig. 5:**
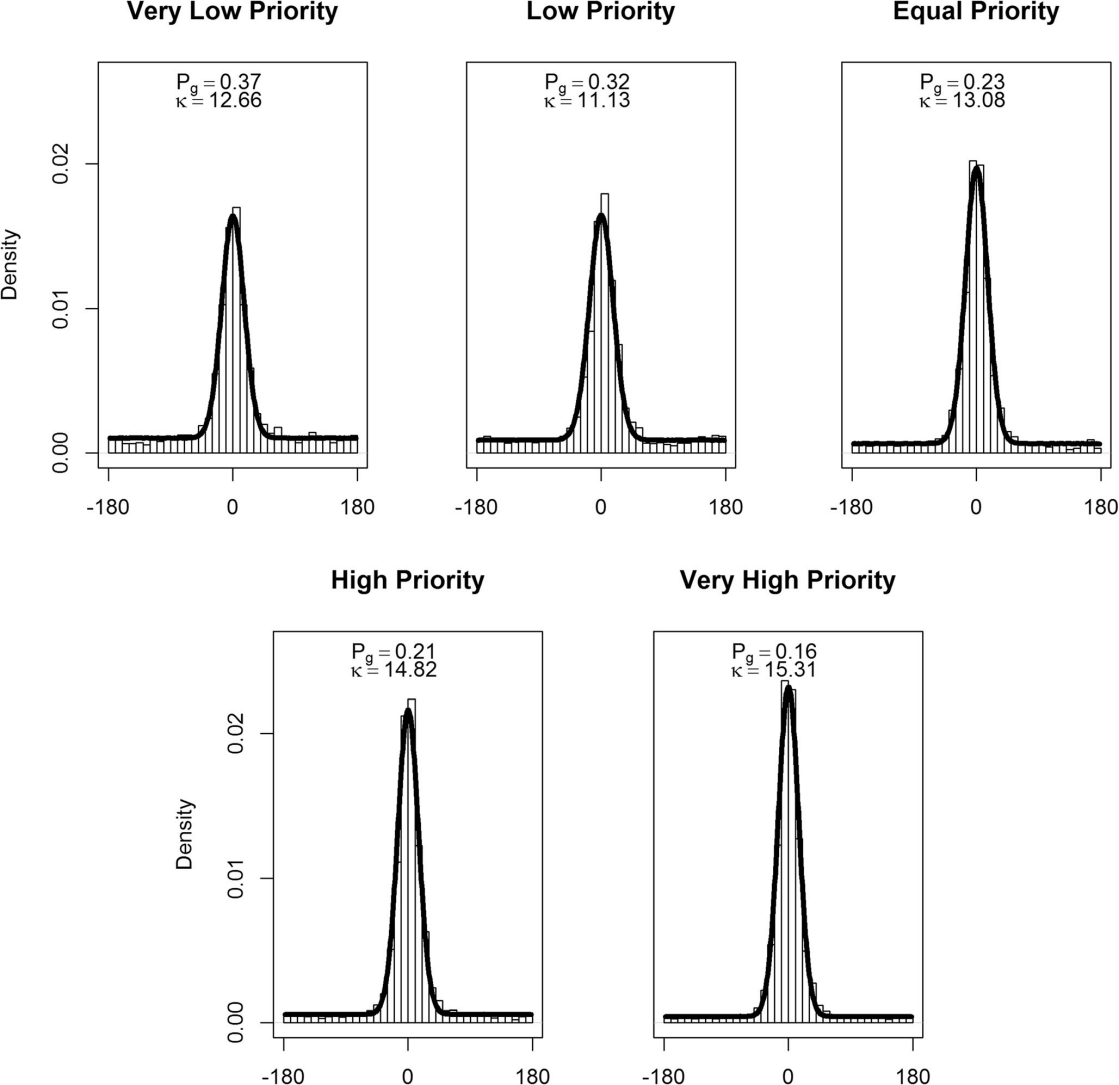
Mixture model fits for all participants for Experiment 2 for each level of target priority. The density plot displays the actual data and the black line shows the model fit. The proportion of guessing (*P*_*G*_) and precision of tracking (*κ*_*VM*_) parameters are also detailed

Figure 5 shows the mixture model fits all the data combined for all participants, for each level of target priority. Fitting the models to each individual participant showed that there was an effect of priority on the proportion of guesses (*P*_*g*_), χ2 (2) = 43.65, *p* < .001 (Fig. 4, right panel). Participants demonstrated less guessing for higher priority targets, *b* = -0.003, SE = 0.001, *t* = 6.85. *Post hoc* comparisons revealed no difference in the proportion of guessing between the very low- and low-priority targets (*b* = -0.001, *t* = 0.39, *p* = .923). Proportion of guessing was higher for the low- compared with equal-priority targets (*b* = -0.006, *t* = 3.58, *p* = .003). However, there was no difference between the equal- and high-priority targets, (*b* = -0.001, *t* = 0.69, *p* = .787). A lower proportion of guessing was revealed in the very high- compared with the high-priority condition (*b* = -0.005, *t* = 4.95, *p* < .001).

There was also evidence for an effect of target priority on the precision of representations *κ*, χ2 (2) = 27.59, *p* < .001, with precision increasing as target priority increased (*b* = 0.15, SE = 0.03, *t* = 5.18) (see Fig. 4, right panel). There was no difference in precision between the very low- and low-, equal- and high-, and high- and very high-priority targets (*t* < 1.86, *p* > .184). There was, however, higher precision in the equal- compared with low-priority condition, *b* = 0.25, *t* = 0.11, *p* = .026.

In line with Experiment 1, the effect of target priority on the magnitude of angular error and proportion of guessing suggests that participants can split attention unequally. Specifically, more attention was allocated to the high-priority target leading to a lower magnitude of error, and overall a lower proportion of guessing. Taken together, this result suggests flexible allocation of the attentional resource and, therefore, does not fit with pure slot-based accounts of attention allocation, which would predict no effect of target priority because, under this account, each target is allocated a single slot.

Experiment 2 explored the extent to which attention splitting is fine-grained, namely the precision with which attention can be divided. There was some evidence for fine-grained spitting because there was a difference in magnitude of angular error and proportion of guessing for the high and very high targets. However, there was no evidence for a difference in these parameters between the very low- and low-priority targets. Since there was only limited evidence for fine-grained splitting, the results cannot distinguish between flexible and hybrid models of attention. No difference in tracking performance between the very low- and low-priority targets could be taken as evidence for a slots + averaging model of attention in which three and one slot(s) were allocated to the high- and low-priority target, respectively, on any given trial, thus resulting in the same pattern of results for both the unequal splitting conditions (i.e., high and low). However, better tracking performance in the very high compared with high pattern fits with a flexible or slots + averaging model, which would predict a graded decrease in magnitude of angular error and proportion of guessing as target priority increases.

Experiments 1 and 2 demonstrated unequal attention splitting in a trajectory-tracking task. Since position tracking does not automatically recruit trajectory-tracking processing during MOT, it has been suggested that position tracking may be a more primary representation during the process of tracking (Howard, Rollings, & Hardie, 2017). To further explore the extent to which unequal attention splitting was possible within a MOT-paradigm, we replicated Experiment 3 using a position-tracking task. This will also potentially provide more insight into the fine-grained nature of unequal attention splitting.

## Experiment 3

Experiment 3 examined whether participants could allocate attention unequally using a different measure of tracking accuracy, to further generalize our findings. Tracking performance in Experiment 3 was indexed by the magnitude of spatial error (we report pixels because we did not standardize viewing distance due to the nature of group testing) from the correct final position of the queried target to the participant’s reported final position of the queried target. More specifically, we used the x, y co-ordinates of the target’s center to index the actual final location and the x, y co-ordinates of the participant’s click to index their position reports. The study aims and hypothesis were preregistered on the Open Science Framework and can be accessed at: https://osf.io/ety5r/?view_only=75f6816e4ade4956a3cdfff270190ca5.

### Method

#### Participants

Forty undergraduate students from the University of Bristol participated in return for course credit. Based on existing data from our lab suggesting an effect size of *dz* = 0.66, we powered for a similar effect size of *d* = 0.5, which gave us at least an 80% chance of observing a similar effect size, with alpha set at .05 for two-tailed tests.

#### Design

Target priority was manipulated in a within-subject design with five levels: very low (30), low (40), equal (50), high (60), and very high (70), and reflected the true probability of a target being queried over the course of the whole experiment. The dependent variable was the magnitude of error (pixels) from the correct final location of the queried target to the participant’s reported final location of the queried target.

#### Procedure

The task was identical to that used in Experiment 2 (group participation condition) apart from the substitution of the trajectory-tracking task with a position-tracking task. At the end of a trial, there was an aural cue that instructed participants to click on the location that they thought the cued target had last occupied. This prompt instructed participants to localize the target (i.e., click the location on the screen where they thought the center of the queried target with the priory stated through the headphones was at the end of the movement). In the 50/50 conditions, the two targets were labelled with either an ‘X’ or a ‘Y’ at the start of the trial, and participants were cued at the end of the trial using these labels. Feedback, consisting of a green disc indicating the correct location of the queried target, was given on each trial for 2,000 ms,d after which the next trial started. Viewing distance was approximately 40 cm.

### Results and discussion

The LME analysis used was identical to Experiment 2. One participant from the analysis because their overall magnitude of error was very high (and the model-based analysis suggested they had very high levels of guessing). All trials on which the size of distance error was greater than 605 pixels were excluded. This value was chosen as it represented the 95th percentile of the data and the density plots showed less uniform responding thereafter.

**Fig. 6:**
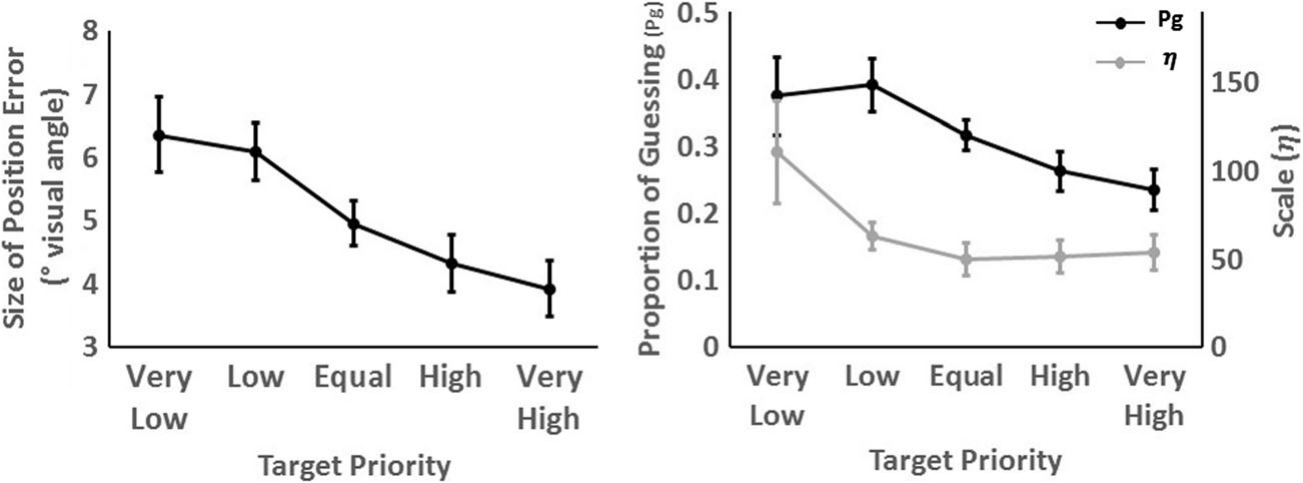
Mean error in size of position error, proportion of guessing and scale of distribution for each target priority in Experiment 3. Error bars represent 95% within-subject confidence intervals using Morey (2008)

There was an effect of target priority on the size of the distance error, χ2 (2) = 67.97, *p* < .001, with distance error decreasing as target priority increased, *b* = -1.75, SE = 0.20, *t* = 8.91. *Post hoc* comparisons showed evidence for smaller distance errors in the high compared with the equal condition, (*b* = -1.67, t = 2.65, *p* = .040), and equal compared with the low priority condition *(b* = -2.99, t = 3.80, *p* = .002) (see Fig. 6, left panel). There was also evidence for smaller error distances in the very high compared with the high condition, (*b* =-1.06, t = 3.10, *p* = .014). There was no evidence for a difference in tracking error between the very low-priority and the low-priority condition, *b* = -0.72, t = 1.31, *p* = .430.

**Fig. 7:**
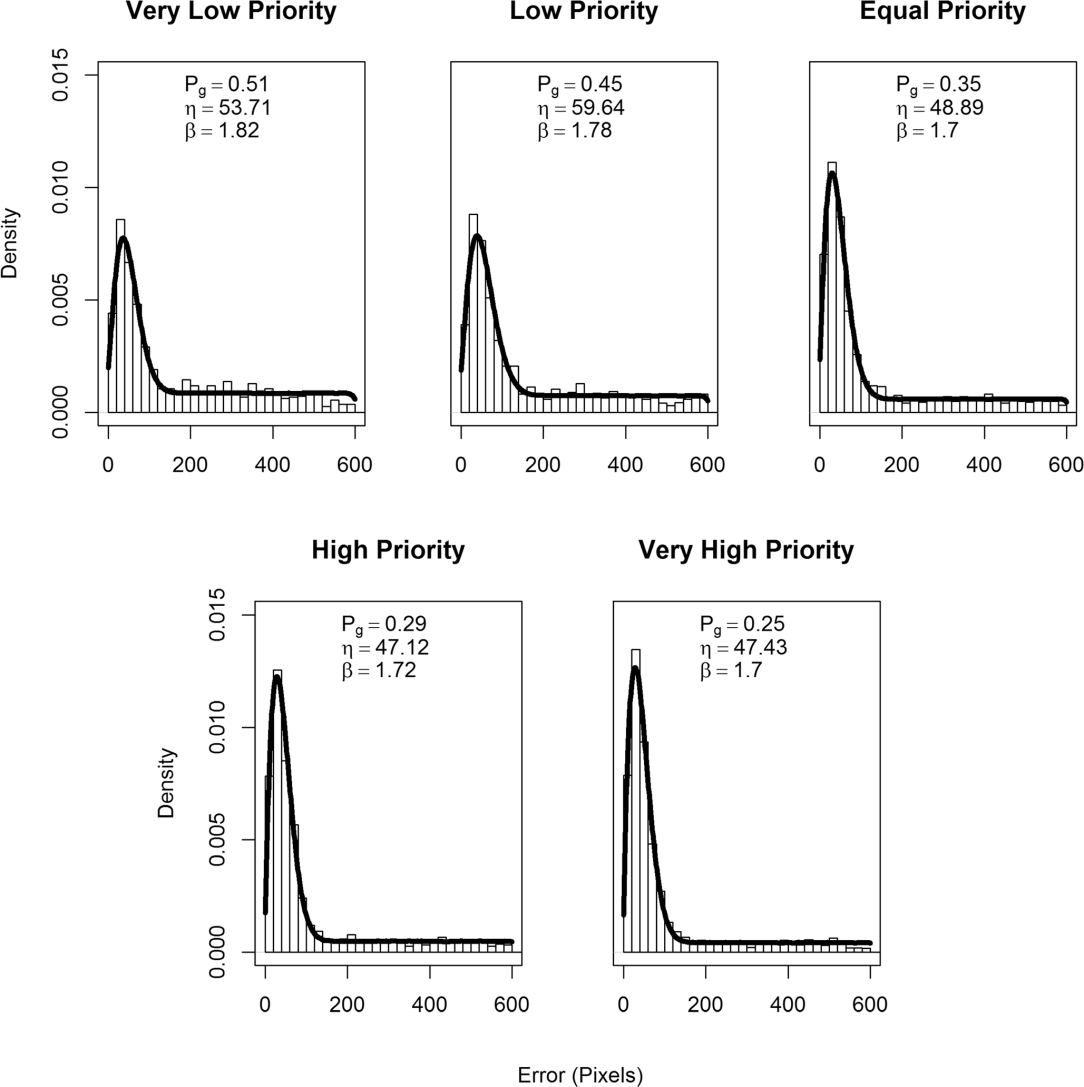
Mixture model fits for all data combined across participants for Experiment 3 for each level of target priority. The histogram plot displays the actual data and the black line shows the model fit. The proportion of guessing (*P*_*G*_) precision of tracking (*β* the Weibull shape), and scale (*η*) parameters are also detailed

In order to fit the data from Experiment 3, we used a different mixture distribution analysis because the error data distribution was linear and positively skewed. We used a Weibull distribution for the tracked items and a uniform distribution (from 0 to 605) for the guessing distribution. The dweibull function used in the analysis of is part of the base distribution package “stats” (R Core Team, 2015). The Weibull has the advantage that both the shape and scale can vary, and can approximate other distributions, including the normal. The error distribution, *ε*, is therefore:

2 $$ \varepsilon ={P}_G{f}_U\left(\mathrm{L}=0,U=605\right)+\left(1-{P}_G\right){f}_{WB}\left(\eta, \beta \right) $$

in which *P*_*g*_ is the guessing rate, L and U are upper and lower bounds for the uniform distribution function *f*_*U*_, and *η* and *β* are the scale and shape of the Weibull distribution function, *f*_*WB*_. Figure 7 shows the mixture model fit to the combined data from all participant for each level of priority. As is evident in the plots, the scale parameter *η* is capturing the spread of the data, which we interpret as the precision of tracked items.

There was an effect of priority on the proportion of guesses, χ2 (2) = 44.18, *p* < .001. Participants demonstrated less guessing for high-priority targets, *b* = -0.004, SE = 0.001, *t* = 6.40 (see Fig. 6, right panel). *Post hoc* comparisons showed evidence for a lower proportion of guessing in the high compared with the equal condition, (*b* = -.005, t = 4.87, *p* < .001), and equal compared with the low-priority condition, *(b* = -.007, t = 3.68, *p* < .003) (see Fig. 6, left panel). There was also evidence for lower proportion of guessing in the very high compared with the high condition, (*b* = -.0.003, t = 2.81, *p* = .027). There was, however, no evidence for a difference in the proportion of guessing between the very low-priority and the low-priority conditions, *b* = .002, t = 0.51, *p* = .819.

There was no evidence for an effect of target priority on the shape, as measured by *β*, of representations, χ2 (2) = 0.71, p = .701. There was, however, evidence for an effect of target priority on scale, as measured by *β*, χ2 (2) = 23.34, p < .001. As target priority increases, the distribution becomes more concentrated, *b* = 1.25, SE = 0.25, *t* = 5.02 (see Fig. 6, right panel). *Post hoc* comparisons revealed evidence for increased concentration for the low- compared with very low-priority condition, *b* = 4.79, t = 3.37, *p* = .007. There was greater concentration in the equal- compared with the low-priority condition, *b* = -1.31, t = 2.75, *p* = .030, and high compared with equal, respectively, *b* = 0.17, t = 1.17, *p* = .004. The distribution for the very high- compared with the high-priority targets was also more concentrated *b* = 0.22, t = 1.15, *p* < .001.

Overall, the position-tracking task revealed evidence for unequal attention allocation that cannot be accounted for by fixed, slot-based models of attention. There was some evidence for more fine-grained attention allocation for the higher priority, with a smaller size of position error and lower proportion of guessing in the very high- compared with the high-priority condition. There was, however, no evidence for fine-grained splitting for the lower priority targets (i.e., 30 vs. 40). This pattern of results is similar to Experiment 2, with participants not differentiating between allocating their attention to a very low- and low-priority target.

## General discussion

In a series of three experiments, we investigated whether participants can split attention unequally to multiple moving objects. Results from all experiments revealed some evidence for unequal attention allocation according to strategic top-down control. This is in line with the existing literature documenting top-down, goal-driven attention allocation in MOT (Brockhoff & Huff, 2016) and visual search (Jiang et al., 2015; Navalpakkam et al., 2010; Shomstein & Johnson, 2013). Such findings replicate research demonstrating unequal attention allocation during MOT in response to instructions (Cohen, Pinto, Howe, & Horowitz, 2011; Yantis, 1992), further supporting the efficacy of using goal-directed instructions to manipulate participants’ attention allocation (Bonnel & Miller, 1994; Fitsoul, 2016).

Across all experiments, the proportion of guessing decreased as target priority increased. Guessing in response to a prompt to report one aspect of a target cannot be equated with a complete withdrawal of attention to all other aspects of that target, since, for example, position and trajectory encoding for targets appear to be distinct processes (Howard, Rollings, & Hardie, 2017). Therefore, for any given modelled guessing response, this may not necessarily indicate a complete withdrawal of attention to that target if its trajectory (Experiments 1 and 2) or its position (Experiment 3) is not known. Even if the participant has completely withdrawn attention from a target, there are two possible reasons that could lead a participant to produce a guess response. They could drop the target (i.e., lose track of it) or swap the target (i.e., confuse it with a distractor). We propose that a combination of these events occur more frequently in the low-priority condition than in the high-priority condition because less attention is allocated to the low-priority target, which constitutes unequal attention allocation. It could be argued that the increased proportion of guessing for low-priority targets compared to high-priority targets reflects participants’ inability to split attention unequally. Specifically, participants may have dropped the low-priority target on some trials and, therefore, on those occasions performed single object tracking, which could be responsible for an increase in the precision for the high-priority target. This is unlikely because the guessing rate and magnitude of error is relatively low across all experiments and indicates non-guessing responses for the lower priority of targets on the majority of trials. Using electrophysiological markers and behavioral experiments, Drew, Horowitz, and Vogel (2013) distinguished between swapping and dropping trials. The relative frequency of these events is not distinguishable in the current data and, therefore, research using such measures within an unequal splitting MOT paradigm is required.

Experiments 2 and 3 assessed how fine-grained unequal attention allocation is. The results from these experiments indicate that, on a given trial, participants can allocate more and less attention to the high- and low-priority targets, respectively. However, the results were less conclusive with regard to how fine-grained such attention splitting is. There was some evidence for fine-grained splitting at higher levels of priority (e.g., between 60 and 70), but not at the lower end of the priority range (e.g., 30 and 40). Perhaps participants could not distinguish between what constitutes 30% and 40% of their attentional resource or were not sufficiently motivated by the task to make the distinction, and so operated according to a binary “more” or “less” mechanism. Alternatively, it is possible that 30% of the attentional resource was sufficient to accurately track the very low-priority targets and therefore that the task was not sensitive enough to distinguish between highly similar target priorities. It is also important to recognize that the response procedure used in our experiments is different to the typical MOT literature in which participants must indicate whether a probed object is a target or a non-target that may have contributed to participants adopting different tracking strategies. However, trajectory and position tracking have been previously shown to be appropriate and sensitive measures of tracking performance that declines with set size (Horowitz & Cohen, 2010; Howard, Rollings & Hardie, 2017).

A persistent debate in the literature surrounds the structure of the attentional resource underlying tracking. Results from all experiments suggest that participants can split attention unequally indicating *some* flexibility to the attentional resource. This does not fit with fixed architecture theories of tracking, which would predict that each target is allocated one slot, and, therefore, there would be no difference in tracking performance. Findings from Experiment 2 and Experiment 3 regarding the fine-grained nature of attention splitting are less conclusive. There is some evidence that participants may be able to only split according to a binary mechanism (i.e., high and low priority) that fits with slots + averaging models, which assume that more than one slot can be allocated to a *high-*priority target. In both the unequal attention splitting conditions (i.e., 30/70; 40/60) three slots and one slot can be allocated to a high- and low-priority target, respectively, and therefore no difference in tracking accuracy is observed. Under this account, no further precision in unequal splitting would be observed, since the slots cannot be subdivided any further, and therefore this model explains the data presented here. Experiment 2 revealed evidence for a difference in magnitude of angular error and proportion of guessing that indicates fine-grained attention allocation. This fits with pure flexible and slots + resources models, which predict a graded increase in tracking-performance measures as target priority increases. Further research is needed to distinguish between these accounts.

Our results fit most closely with hybrid models of attention allocation. Pure flexible accounts require an additional assertion that not only can the resource be divided in a fine-grained manner, but that this fine-grained allocation of the resource can be divided out *unequally* between targets. A relevant analogy here might be the division of pay between workers: if 40 units (dollars, euros, etc.) of currency are to be shared between four workers, the fixed account would suggest that there are four ten-unit notes, which can be shared out, where a flexible account would suggest that there are in fact 4,000 subunits (e.g., cents) to be shared out. The flexible account asserts that this sum could be divided amongst 4,000 workers (actually an infinite number, but this requires subdivision of cents into electronic payments of less than 1 cent for the purpose of this analogy). However, the flexible account has so far been silent on whether or not this payment could be made unequally between workers, with some receiving more than others. The evidence we present here suggests that this is the case, that attention can be flexibly *and unequally* divided. How this unequal splitting of attention is achieved by the visual system does, however, warrant further theoretical consideration in the MOT literature.

The guessing rate remained relatively low throughout; indeed, the mean guessing rate for the lowest priority targets across Experiments 2 and 3 was 29%. This is important because it suggests that on the majority of trials, participants did not appear to adopt the strategy of only single-object tracking of the high-priority target, in which case we might expect nearer a 100% guess rate for the lower priority target. However, the results reported were averaged across trials and so it is possible that participants did not attempt to track *multiple* objects on each and every trial. Specifically, it is possible that participants engaged in single-object tracking and used target priority to determine the number of trials on which they tracked *only* the high- or low-reward target. However, this is unlikely because there were only two targets, which is below the proposed four-object capacity limit for tracking. Whereas examining within-trial behavior was not the main focus of this article, future research should focus on *how* participants achieve this unequal splitting. One way to directly investigate this would be by probing both targets at the end of a trial to gain insight into the relationship between tracking accuracy on the two simultaneously presented targets. A positive correlation between tracking performances would indicate that participants were engaging in multiple-object tracking because performance on a given trial is broadly either *good* or *bad* for both targets*.* A negative correlation would indicate that participants were engaging in single-object tracking because, as accuracy on one target (i.e., the tracked target) increases, accuracy on another target (i.e., the untracked target) decreases. No correlation between performance on the two targets might be consistent with participants attention fluctuating within a trial and, therefore, tracking a single object at the cost of another.

Although the studies presented indicate unequal attention allocation when performance is examined at the *trial level*, it is not possible to determine participants’ attention allocation during the trial. It is possible that participants were tracking one target at a time but switched between targets during the trial, spending relatively more time on higher priority targets. Some have argued that attention is flexibly allocated in experiments investigating stimulus-driven unequal attention allocation (e.g., Iordanescu, Grabowecky, & Suzuki, 2009). Therefore, it is possible that prioritization and unequal attention allocation only occurs when, for example, tracking becomes difficult as in response to reduced inter-object spacing (Meyerhoff, Schwan, & Huff, 2018). Future research is therefore required to examine how attention is allocated at different points within a trial. One possible avenue is to use a dot probe detection task (e.g., Meyerhoff, Schwan, & Huff, 2018) in which probes are randomly presented within the tracking phase or two lateralized tracking areas are utilized to index attention allocation at different timepoints in a trial. Such research will also provide detail into the interplay between stimulus-driven and goal-directed attentional mechanisms within MOT.

A further consideration of the tasks we have used is that equal and unequal attention splitting are potentially different tasks. Traditional MOT tasks might best be characterized primarily as an equal attention-splitting task, although some have argued for unequal attention splits and attention reallocation in MOT (e.g., Iordanescu et al., 2009). The unequal attention-splitting MOT task used in these experiments also has a multiple identity-tracking component because participants must assign a target priority (a form of identity) to each of the targets. Identity encoding is not automatic during MOT (Pylyshyn, 2004; Scholl & Pylyshyn, 1999) and has been shown to require resources (Cohen, Pinto, Howe & Horowitz, 2011), in part due to identity-location binding processes (Saiki, 2002; Oksama & Hyönä, 2008). Future research should examine whether attention can be divided unequally in a purer MOT paradigm that does not require identity-location bindings. For example, distinct tracking areas or “cages” (e.g., Howard and Holcombe, 2008) could be presented on each trial and each tracking area would be associated with a certain likelihood of being probed. This design would not require participants to maintain identity-location bindings because there would only be one target in each tracking area with, for example, three distractors.

These data demonstrate that participants can split attention unequally in MOT tasks. There is, however, limited evidence that this ability is fine-grained. These findings are not consistent with fixed, slot-based accounts of attention allocation. Pure flexible accounts could account for the results, but with the additional assumption that attention may be divided unequally between targets. Hybrid models, specifically the slots + averaging model, explain the data reported here without further assumptions. Since these models have traditionally been applied to memory tasks, similar models specific to MOT that can account for the flexibility of attention demonstrated here, are required.

## Footnotes

1 We thank H. Meyerhoff for suggesting this analysis.

2 The same qualitative pattern of results was observed when each experiment was analyzed independently. When experiment was included as a between-subject factor there were no reliable differences. Note, under a Bayesian framework combining the data is equivalent to multiplying the Bayes factors from each experiment (assuming the posterior from Experiment 1 is the prior for Experiment 2; see Ly, Etz, Marsman, & Wagenmakers, 2018).

3 We did not include analysis of this aspect of the design as so few participants actively engaged with it, but note the same qualitative pattern of results was observed when “untracked” trials were excluded.
